# *Babesia microti*—*Borrelia burgdorferi* Coinfection

**DOI:** 10.3390/pathogens8030117

**Published:** 2019-07-31

**Authors:** Nikhat Parveen, Purnima Bhanot

**Affiliations:** Rutgers New Jersey Medical School, Department of Microbiology, Biochemistry and Molecular Genetics, Newark, NJ 07103, USA

**Keywords:** *Babesia*, Borrelia, Babesiosis, Lyme disease, coinfection, tick-borne pathogens

## Abstract

The incidence and geographic distribution of human babesiosis is growing in the U.S. Its major causative agent is the protozoan parasite, *Babesia microti*. *B. microti* is transmitted to humans primarily through the bite of *Ixodes scapularis* ticks, which are vectors for a number of other pathogens. Other routes of *B. microti* transmission are blood transfusion and in rare cases of mother-to-foetus transmission, through the placenta. This review discusses the current literature on mammalian coinfection with *B. microti* and *Borrelia burgdorferi*, the causative agent Lyme disease.

## 1. Babesiosis is an Important Tick-Borne Disease

The major cause of human babesiosis in the U.S. is the tick-borne protozoan parasite, *Babesia microti*. *B. microti* sporozoites are transmitted to mammalian hosts through the bite of *Ixodes scapularis* ticks. Sporozoites invade erythrocytes and within them develop into trophozoites and merozoites. Once released, merozoites infect more erythrocytes. The repeated rounds of erythrocytic invasion cause symptoms ranging from a mild flu-like fever to severe anemia, multiorgan failure, and death [1]. Immunocompetent individuals are generally asymptomatic but immunodeficiencies resulting from asplenia, B-cell lymphomas, organ transplants, HIV/AIDS, or treatment with immunosuppressive drugs, such as Rituximab, heighten the risk of babesiosis [1].

Babesiosis is endemic in the northeastern and upper midwestern U.S. [2], and has been spreading to new regions as the geographic range of *Ixodes* ticks expands [3,4,5,6,7]. Since being added to the list of nationally notifiable diseases in 2011, the number of babesiosis cases reported to the Centers for Disease Control and Prevention (CDC) has more than doubled, from 1124 in 2011 to over 2300 in 2017, and the number of states reporting babesiosis has increased from 18 to 31 [8] (Centers for Disease Control and Prevention, National Notifiable Diseases Surveillance System, 2017 Annual Tables of Infectious Disease Data). The number of cases is likely an underestimate since diagnosing babesiosis can be challenging: Early clinical manifestations are non-specific and require a high index of suspicion.

## 2. *B. microti* is the Most Common Transfusion-Transmitted Pathogen in the U.S.A.

While human babesiosis is primarily a zoonotic disease, human-to-human transmission can occur through transfusion of contaminated blood products [9]. Transfusion-transmitted babesiosis has a mortality rate of 20% [10]. This poses a major hazard to the supply of safe blood/blood products because infected, asymptomatic blood donors are unaware of their status at the time of donation. In rare cases, *B. microti* can also be transmitted vertically [11,12,13,14] and through organ transplants in humans [15]. Although rare in humans, transplacental transmission of *B. microti* occurs at high frequencies in its natural enzootic and reservoir hosts [16,17].

Treatment regimens for babesiosis are limited by the emergence of drug-resistant parasites, toxicity, and failure of drugs [4,18,19]. The confluence of rising disease incidence, expanding vector range, difficulty in early diagnosis, lack of mandatory donor screening, and drawbacks of current therapies make babesiosis an important emerging threat to human health.

## 3. *B. microti*—*Borrelia burgdorferi* Co-Infection is Common in Vector and Host

The tick vector for *B. microti*, *Ixodes scapularis,* also carries a number of other human pathogens: *Borrelia burgdorferi* (henceforth *Bo. burgdorferi*, the causative agent of Lyme disease), *Anaplasma phagocytophilum* (the causative agent of anaplasmosis), *Borrelia miyamotoi* (the causative agent of *B. miyamotoi* disease), and Powassan virus (the causative agent of deer tick encephalitis) [20]. Of these, *Bo. burgdorferi* causes significant public health concern due to the high disease burden. *Bo. burgdorferi* is a spirochete that remains extracellular in the mammalian host. Its ability to disseminate from the site of bite in the skin to peripheral organs is strain-dependent. Virulent strains cause symptoms ranging from arthritis and carditis to neurological manifestations [21]. Depending on the collection site and the life-stage of the tick, the percentage of ticks coinfected with *B. microti* and *Bo. burgdorferi* ranges from 0%–13% [22,23,24,25]. Coinfections in the natural and reservoir hosts are also prevalent [26]. Simultaneous infections can occur through transmission of multiple pathogens by the same tick [27] or temporally independent transmission of different pathogens by different ticks. Disease manifestations may be unaffected, potentiated or attenuated by coinfections.

Serological studies indicate that coinfection with *B. microti* and *Bo. burgdorferi* is common in humans [28]. In endemic regions, almost 20% of Lyme disease patients reported concurrent babesiosis while up to 25% of babesiosis patients also had Lyme disease [22]. A large percentage of patients with chronic/post-treatment Lyme disease syndrome (52%) show evidence of past or active *Babesia* coinfection [29]. Since antibiotics used to treat *Bo. burgdorferi* infection are ineffective against *B. microti* and diagnosis can be challenging, human coinfection with *B. microti* and *Bo. burgdorferi* is of significant clinical concern.

While this review focuses on coinfection with *B. microti* and *Bo. burgdorferi*, there is some evidence that coinfections with a different *Babesia* species, *B. duncani*, and *Bo. Burgdorferi* may be more common than previously suspected. *B. duncani* was thought to be restricted to the west coast of the U.S. but recent reports find evidence of potential *B. duncani* infections in the northeastern U.S. [29]. Since *B. duncani* is widespread in Canada [30], its southern spread into northeastern U.S., an area already endemic for Lyme disease, makes coinfections with *B. duncani* and *Bo. burgdorferi* a possibility that needs to be carefully investigated. Antibiotics used against *B. microti* are less effective against *B. duncani* [31], making treatment of *B. duncani* potentially more challenging.

The effect of coinfection by the two pathogens on the host is debated. Some human patient data suggest that coinfection or exposure to both pathogens could lead to complicated outcomes. Of the two studies that have examined the effect of *B. microti*—*Bo. burgdorferi* coinfection on humans, one found worse symptoms in patients infected with *B. microti* alone compared to patients with exposure to both pathogens [32]. However, the second study reported no difference in the number or duration of babesiosis symptoms between the two groups [33]. In both studies, coinfected patients reported symptoms of greater variety and longer duration compared to patients infected with *Bo. burgdorferi* alone [32,33]. The long-term clinical outcomes of the two groups were similar. A major challenge of human studies is that it is often not possible to determine if patients were infected concomitantly or serially with the two pathogens. It can also be difficult to distinguish active infection from past exposure based exclusively on serological testing.

Timing of infection can be controlled in animal studies. Experimental coinfections in rodents can provide insights into pathophysiologial processes that are useful for understanding human disease. A few investigations have examined the reciprocal interaction between *B. microti* and *Bo. burgdorferi* using laboratory mouse strains [34,35,36]. One study examined *B. microti’s* effect on carditis and arthritis induced by *Bo. burgdorferi*. It found that arthritis severity, as measured by histopathological examination, was enhanced in coinfected Balb/c mice compared to mice infected with *Bo. burgdorferi* alone, while carditis severity was similar in both groups [36]. Similar results were obtained in C3H/HeJ mice by our group [34,37].

A second study examined the effect both of *B. microti* on *Bo. burgdorferi*-associated symptoms and of *Bo. burgdorferi* on *B. microti*-associated pathology [35]. This study examined the effect of coinfection in asplenic, young and old mice since the asplenia and old age are risk factors for human babesiosis. It found *B. microti* peak parasitemia trended higher in young C3H/HeN mice infected with *B. microti* alone compared to the coinfected cohort, but the difference did not reach statistical significance. Histopathological scores for arthritis trended higher in coinfected mice, compared to *Bo. burgdorferi*-infected mice as well, but did not reach statistical significance. Interestingly, inflammation scores for carditis were lower in coinfected mice compared to mice infected with *Bo. burgdorgferi* alone but again, the difference was not statistically significant. For this reason, the study concluded that the two diseases follow independent courses in a mouse model of coinfection [35]. Our recent work suggests that there may be interaction between the two infections. We found that peak *B. microti* parasitemia was consistently and significantly lower in young, coinfected C3H/HeJ mice compared to parasitemia in mice infected solely with *B. microti* [37]. In addition, arthritis severity was significantly higher in coinfected mice than in mice infected solely with *Bo. burgdorferi* [37]. On balance, existing data suggests exacerbation of Lyme arthritis by *B. microti* and attenuation of *B. microti* parasitemia by *Bo. burgdorferi*, at least in coinfected C3H mice.

The interaction between *B. microti* and *Bo. burgdorferi* in their natural rodent host, *Peromycus leucopus,* is of great importance to the ecological epidemiology of human babesiosis since it could impact the geographic spread of the two pathogens. A pioneering study investigated transmission of *B. microti* and *Bo. burgdorferi* to ticks and also examined the effect of *Bo. burgdorferi* coinfection on *B. microti* parasitemia in laboratory-bred *P. leucopus* that were infected by tick bite [38]. It found higher parasitemia in coinfected mice compared to *P. leucopus* infected with *B. microti* alone. These results are in contrast to ones obtained from infections of laboratory mouse strains where coinfection had either a neutral or a depressive effect on *B. microti* parasitemia. There are several explanations for these seemingly contradictory results. First, this study used a strain of *Bo. burgdorferi* that, in mice, is slow to disseminate from the site of infection [39]. Non-invasive strains of *Bo. burgdorferi* elicit a different set of host responses, which might impact *B. microti* differently, from ones elicited by invasive strains of *Bo. burgdorferi* used in previous murine coinfection studies [40,41]. Second, this study infected *P. leucopus* through tick bite. While tick bite has the notable advantage of being the natural route of transmission for both pathogens, it introduces the confounding variable of unequal inoculum dose. In experimental infections, *I. dammini* (renamed *I. scapuralis* in 1996 [42]) nymphs infected with *B. microti*-infected blood, as larvae, infected only 50% of hamsters over the course of 54 h [43]. Similarly, infection studies of *Plasmodium berghei*, a vector-borne protozoan parasite related to *B. microti*, have demonstrated a wide variance in the number of organisms transmitted through vector bite: Approximately 10%–20% of feeding mosquitoes, despite being infected, did not inject any *P. berghei* into the mouse host [44,45]. The inoculum size is an important consideration since *P. berghei* sporozoites in the skin trigger a dermal immune response that has long-lasting impact on the subsequent infection by sporozoites of hepatocytes [46]. By analogy to the above-mentioned transmission dynamics of *P. berghei* through mosquito bites, infection of *B. microti* and *Bo. burdorferi* through tick bites could introduce different numbers of pathogens into the two cohorts of mice. Potential differences in inoculum make it difficult to directly compare parasitemias between different groups of bite-infected mice. Transmission through tick-bite also introduces tick salivary gland factors that serve multiple functions that assist in pathogen transmission, including vasodilation, anti-coagulation, and fibrolysis (for a recent review refer to [47]). If there are salivary gland proteins specifically associated with *B. microti* and/or *Bo. burgdorferi* during transmission, then a local host immune response induced by them could impact early steps of infection by both pathogens. There is a gap in our understanding of the effects of saliva on *B. microti* transmission in the presence and absence of *Bo. burgdorferi* and other pathogens. Third, as a consequence of transmission through tick bite, *B. microti* infection in this study was initiated by sporozoites (the stages introduced by tick bite) rather than infected erythrocytes, as done in previous studies. Early immune responses elicited during a sporozoite-initiated infection could have kinetics and cellular characteristics that are different from that elicited by intravenous or intraperitoneal injections of *B. microti*-infected erythrocytes. *B. microti* sporozoites and erythrocytic stages are highly likely to be immunologically and biologically distinct. Finally, interaction between *B. microti* and *Bo. burgdorferi* may be different in *P. leucopus* and *Mus muculus* (C3H) mice. Clearly, additional studies are needed to test the interplay of host species with (1) *B. microti* and *Bo. burgdorferi* genotypes, (2) *B. microti* sporozoite-initiated and infected red blood cell (RBC)-initiated infections, (3) transmission through injection versus tick-bite.

A corollary to higher *B. microti* parasitemia in coinfected *P. leucopus* mice was the suggestion that *Bo. burgdorferi* facilitates the geographic spread of *B. microti* [38], which is otherwise acquired by ticks less efficiently compared to *Bo. burgdorferi* [48]. This model was proposed prior to the discovery of *B. microti’s* highly efficient transplacental transmission in *P. leucopus* [18]. *B. microti’s* vertical transmission is likely to be an important vector-independent contributor to the geographic expansion of *B. microti*. This mode of transmission will be promoted by warmer winters brought about by global climate change and could be an additional explanation for the expansion in range by *B. microti.*

## 4. Model for *B. microti’s* Effect on *Bo. burgdorferi* in C3H Mice

**Figure 1:**
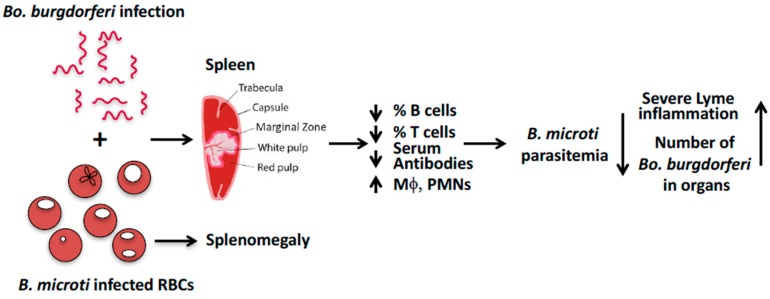
A model to explain the effect of B. microti and Bo. burgdorferi coinfection in M. musculus.

We suggest that in C3H mice, *B. microti* coinfection exacerbates symptoms of arthritis caused by *Bo. burgdorferi* because *B. microti* causes splenic dysfunction that reduces B- and T-cell function and the production of antibodies required to control *Bo. burgdorferi* infection (Figure 1) [37]. The resulting increased survival of *Bo. burgdorferi* in coinfected mice, compared to mice infected with *Borrelia* alone, enhances inflammatory Lyme arthritis in coinfected mice. Reciprocally, *Bo. burgdorferi* infection triggers TLR2-based signaling (mainly due to its lipoproteins) that increases pro-inflammatory cytokines, and activates macrophages and polymononuclear neutrophils. These innate immune responses diminish *B. microti* parasitemia in coinfected mice compared to mice infected only with *B. microti*.

The inconsistent outcomes of different human and animal studies point to the need for further investigations. A murine model of human coinfection enables control of inoculum timing and size, larger sample sizes, and mechanistic studies. Drawbacks are that a mouse model does not recreate all aspects of human disease, for example, while advanced age is a risk factor for human babesiosis, in mice it confers resistance to *B. microti* infection [34,49]. Nonetheless, judicious use of animal models to define specific pathophysiological processes of human diseases can complement studies of human patients. Murine studies may need to employ large sample sizes to ensure adequate power to reveal small but biologically meaningful differences between coinfected and single-infected animals. These studies need to determine if the reciprocal interaction between the two pathogens is affected by the invasiveness of the *B. microti* and *Bo. burgdorferi* strains, different mouse species (*Mus musculus* versus *P. leucopus*), the route of transmission (intradermal, intravenous, and tick bite), infection by *B. microti* sporozoites or infected RBCs, and the relative timing of infection (concomitant versus sequential). Finally, host sex has to be considered as a biological variable. Two studies found that male mice were more susceptible to infection with *B. microti* [50,51] and one study found female mice were more susceptible to infection with *B. duncani* (WA-1). A clarification of the role of host sex in *Babesia* infections will shed light on whether the higher prevalence of human babesiosis amongst men (Surveillance for Babesiosis—United States, 2014 Annual Summary. Atlanta, Georgia: U.S. Department of Health and Human Services, CDC, 2016) is a result of higher environmental exposure and/or increased susceptibility of males to *B. microti*.
